# Impact of delayed pelvic imaging on the staging of biochemical recurrence in prostate cancer patients using [^18^F]DCFPYL PET/CT: a retrospective evaluation

**DOI:** 10.1186/s41824-025-00238-8

**Published:** 2025-02-01

**Authors:** Ana Rodríguez-Pajuelo, Miriam Guerra-Gómez, Juan Ignacio Cuenca Cuenca, José María Freire-Macías, José Manuel Jiménez-Hoyuela García, Rosa María Álvarez-Pérez

**Affiliations:** Nuclear Medicine Department, University Hospital Virgen del Rocío, Seville, USA

**Keywords:** Prostate cancer, Dual-phase, PSMA-PET, [^18^F]DCFPYL, PSMA-RADS.

## Abstract

**Purpose:**

The aim of this study was to evaluate the added diagnostic value of additional second stage pelvic scanning as part of the [^18^F]DCFPYL PET/CT procedure in patients treated for prostate cancer (PCa) who have biochemical recurrence (BR).

**Materials and methods:**

Consecutive patients with a diagnosis of PCa who underwent a dual-phase PSMA-PET scan between September 2022 and December 2023, were retrospectively included. We analyzed the number and maximum SUV (SUVmax) of lesions only in the pelvic region (prostate, locoregional lymph nodes and bone), based on PSMA-RADS version 2.0 and miTNM criteria. To assess the potential diagnostic benefit of additional delayed pelvic PET/CT imaging as part of the PSMA-PET procedure, the change in molecular TNM classification was evaluated after the procedure.

**Results:**

Additional delayed pelvic PET/CT imaging as part of the PSMA-PET procedure resulted in a change in molecular TNM classification in 22 out of 136 patients (16.2%). The highest percentage change was obtained in the miN classification (14/22 patients), followed by the miT classification (7/22) and lastly miM (1/22). Moreover, we found that patients in whom delayed pelvic imaging resulted in a change in molecular TNM classification were significantly older and had a higher PSA level than those in whom delayed imaging did not provide additional information.

**Conclusions:**

Pelvic delayed imaging in patients with biochemical recurrence of prostate cancer undergoing PET/CT with [^18^F]DCFPYL shows a non-negligible influence on patient staging, modifying the miTNM classification in 16.2% of cases, with pelvic lymphatic involvement benefiting the most from the dual study.

**Supplementary Information:**

The online version contains supplementary material available at 10.1186/s41824-025-00238-8.

## Introduction

In the last decade, positron emission tomography/computed tomography (PET/CT) with prostate-specific membrane antigen (PSMA) has become the preferred diagnostic test of choice for restaging patients with biochemical recurrence (BR) of prostate cancer (PCa) ((Trabulsi et al. 2020). Among the many drugs developed in PET imaging, [^18^F]DCFPYL (2-(3-{1-carboxy-5-[(6–18 F-fluoro-pyridin-3-carbonyl)-amino]-pentenyl}-ureido)-pentanodioic acid) has shown great utility in the evaluation of these patients (Morris et al. 2021; Song et al. 2020; Wondergem et al. 2017; Rowe et al. 2016). 

However, identification of recurrence in PCa patients is particularly limited by urinary excretion of the tracer, which may reduce accuracy when residual disease is in the prostate bed or periureteral lymph nodes ((Bauckneht et al. 2023). An alternative for future studies could be the use of [^18^F]F-PSMA-1007 which is excreted hepatically rather than renally and may have better diagnostic efficacy for detecting local recurrence in the bladder area ((Giesel et al. 2017).

To date, few publications have assessed the diagnostic value of dual imaging in the [^18^F]DCFPYL PET/CT procedure with an acquisition at 60 min post-radiotracer administration and a delayed image obtained at 120–180 min. Implementing a delayed image faces clinical challenges, such as patient compliance, scheduling and PET/CT workflows ((Bauckneht et al. 2023; Koehler et al. 2022; Morawitz et al. 2022).

The latest EANM/SNMMI ((Fendler et al. 2023) guideline recommends imaging 60 min after radiopharmaceutical injection, leaving second-stage abdomino-pelvic imaging as an optional procedure.

Therefore, the combined protocol with additional delayed imaging is not standardised in the clinical setting and its added clinical value still needs to be clarified ((Alberts et al. 2021).

The aim of this study was to evaluate the added diagnostic value of additional second stage pelvic scanning as part of the [^18^F]DCFPYL PET/CT procedure after bladder emptying, in patients treated for PCa who have BR.

## Materials and methods

### Patients

Consecutive patients with a diagnosis of PCa, who were being studied for suspected BR or for restaging in selected high-risk cases, who underwent a dual-phase PSMA-PET scan between September 2022 and December 2023, were retrospectively included.

The study was conducted in accordance with the regulations of the Hospital Ethics Committee in line with the principles outlined in the Declaration of Helsinki.

### PSMA-PET procedure

No fasting was required, and no diuretics were administered prior to imaging. Images were acquired using a Discovery MI 5R digital PET/CT machine (General Electric Healthcare, USA) following intravenous injection of a standard dose of 314.5 MBq of [^18^F]DCFPYL (Pylclari^®^ CuriumPharmaSpain).

Within 60 min after injection, a first image was acquired from the middle third of the femur to the vertex, with an acquisition time of 3 min/bed. An image acquisition of the pelvis was systematically performed within 120 min, with an acquisition time of 5 min/bed. A low-dose CT acquisition was used with the following settings: voltage 120 KV reference current 130 mAs and slice thickness 3.75 mm.

PET images were reconstructed with a Bayesian penalised likelihood Q.Clear^®^ (Discovery MI) reconstruction algorithm with a beta factor of 600 for both early-phase and delayed-phase images. CT attenuation correction was performed in all patients. Scans were analysed with Hermes Medical Solutions software.

### Image interpretation

The images were evaluated independently by two experienced nuclear physicians. Discrepancies were resolved in a consensus reading. The readers were blinded to the clinical data of the patients.

We included in the analysis the location of lesions in the pelvic region only, both in early and second-stage imaging, analysing number and maximum SUV (SUVmax) of lesions for each of the pelvic regions (prostate, locoregional lymph nodes and bone), based on PSMA-RADS version 2.0 and miTNM criteria ((Rowe et al. 2018; Eiber et al. 2018). 

To measure background SUVmax, a region of interest was placed in the left gluteus maximus muscle on early and delayed phase PET/CT.

For each pelvic region the following values were calculated:

- SUVratio early = SUVmax early / SUVmax gluteus.
- SUVratio delay = SUVmax delay/ SUVmax gluteal.
- ΔSUVmax = SUVmax early – SUVmax delay.
- SUVmax IR = (ΔSUVmax / SUVmax early) x 100.
- SUVmax relation = SUVmax delay / SUVmax early.
- ΔSUVratio = SUVratio early –SUVratio delay.
- SUVratio IR = (ΔSUVratio / SUVratio early) x 100.
- SUVratio relation = SUVratio delay / SUVratio early.

In addition, [^18^F]DCFPYL uptake deposits were also qualitatively analysed, differentiating lesions into malignant or benign, using as reference standard histopathology when available, or follow-up imaging with PSMA-PET, bone scan, CT, MRI and/or PSA decline at 6 months after treatment. Furthermore, a decrease in PSA value during follow-up in those patients in whom active surveillance was chosen was also considered as a reference standard.

Malignant lesions were considered to be PCa-related if one of the following criteria was met: histopathology of the lesion showed adenocarcinoma of the prostate, follow-up imaging showed an increase in size or number of lesions or a decrease in size or number of lesions after treatment (MRI, CT, Bone scan or [^18^F]Pylclari PET/CT), and when there was an increase in PSA during follow-up or a decrease in PSA in response to treatment. If the above conditions were not met, they were determined to be benign lesions.

To assess the potential diagnostic benefit of additional delayed pelvic PET/CT imaging as part of the PSMA-PET procedure, the change in molecular TNM classification was evaluated after the procedure.

### Statistical analysis

Categorical variables were expressed as frequency and percentage and analysed using the χ2 test or Fisher’s exact test. Continuous variables were expressed as mean and standard deviation (SD) or median and interquartile range (IQR).

For quantitative variables, normal distribution was assessed with the Saphiro-Wilk test. Student’s t-test and its non-parametric alternative, the Mann-Whitney U-test, were used to study mean differences between groups. For the different statistical tests, 95% confidence intervals were calculated for quantitative variables, and Odds Ratio [95% CI] for qualitative variables. Significant differences were determined to exist when *p* < 0.05.

Statistical analysis was calculated using IBM SPSS^®^ Statistics version 24.

## Results

A total of 136 patients undergoing dual-phase PSMA-PET with [^18^F]DCFPYL were included, of which 124 (91.2%) were being studied in the context of biochemical recurrence, while in 12 patients (8.8%) the study was requested for initial restaging. The baseline characteristics of the patients are shown in Table 1.

**Table 1:** Baseline patient characteristics

Patient characteristics (*n* = 136)	
Age (years)*	66.5 (6.7) (49–84)
**Gleason (n**,** %) [1]**	
**4**	1 (0.7%)
**5**	2 (1.5%)
**6**	30 (22.2%)
**7**	66 (48.9%)
**8**	24 (17.8%)
**9**	11 (8.1%)
**10**	1 (0.7%)
PSA at the time of PSMA-PET (ng/ml)**	0.45 (0.7) (0.01- 4,3)
**Clinical justification PSMA-PET (n**,** %)**	
**Initial staging**	12 (8.8%)
**Biochemical recurrence**	124 (91.2%)
**Reference standard (n**,** %)**	52 (38.2%)
**Histopathology**	1
**Follow-up images**	
MRI	1
CT	14
Bone scan	7
PSMA-PET	18
**PSA after treatment**	32
**Follow-up PSA**	7

*Quantitative variables expressed by mean (SD) and range (minimum - maximum). ** Quantitative variables expressed as median (IQR) and range (minimum - maximum). [Missing values]

The mean age was 66.5 years (SD 6.7; range: 49–84 years). Most patients had a Gleason score equal to or greater than 7 (*n* = 102/136; 75%), with Gleason 7 being the predominant score (*n* = 66/136; 48.9%).

Prostate lesions were detected in a total of 16/136 patients (11.8%), pelvic lymph nodes in 26/136 patients (19.1%) and pelvic bone lesions in 3/136 patients (2.2%).

In early-phase PET/CT, the SUVmax for local recurrences, pelvic lymph node metastases and pelvic bone metastases was 12.75 ± 11.7, 26.35 ± 42.6 and 4.54 ± 114.9, respectively, while delayed-phase SUVmax was 17.9 ± 15.6, 32.9 ± 47.7 and 5.35 ± 127.9 respectively, there being no statistically significant difference between them (*p* = 0.221). There were also no differences in SUVmax on delayed-phase PET/CT between prostate lesions and pelvic lymph nodes, as well as in ΔSUVmax, IR and ratio values (Table 2).

**Table 2:** Distribution of SUVmax and SUV ratio values and the different SUV coefficients in malignant lesions

	SUV maximum					
	SUVmax early	SUVmax delay	ΔSUVmax [CI 95%]	SUVmax IR	SUVmax relation	Differences†
**Prostate lesions** (***n*** = **16**)	12.75 (11.7) (3.85–52.9)	17.9 (15.6) (7-70.4)	1.5 (0.4) (0.98–2.2) [CI -7.85; -2.44]	-5,15 (5,1) (-17,45 − 0,25)	-50.77 (39.58) (-120.11–1.89)	*p* = 0.001
**Pelvic lymph nodes** (***n*** = **26**)	26.35 (42.6) (1.3–169)	32.9 (47.7) (1.84–183)	-6.6 (7.2) (-29.1-0.99) [CI -9.51; -3.67]	-57,5 (68,4) (-280-26,83)	1.57 (0.68) (0.73–3.8)	*p* < 0.001
**Differences**	*p* = 0.221	*p* = 0.231	*p* = 0.489	*p* = 0.724	*p* = 0.724	
	**SUV ratio**					
	**SUVratio early**	**SUVratio delay**	**ΔSUVratio** **[CI 95%]**	**SUVratio IR**	**SUVratio relation**	**Diferencias**†
**Prostate lesions** (***n*** = **16**)	12.3 (8.5) (3.28–37.8)	21.9 (15.46) (5.8–70.4)	-8.89 (8) (-32.56 – (-1.54)) [CI -13.1; -4.7]	-81.97 (56.27) (-224.9 – (-14.61))	1,81 (0.56) (1.15–3.25)	*p* < 0.001
**Pelvic lymph nodes** (***n*** = **26**)	61.3 (46.3) (1.4-178.4)	37 (50.6) (1-179.4)	24.3 (68.5) (-113-177) [CI -3.34;51.96]	-94.25 (591) (-2955-99.44)	1.9 (5.9) (0.01–30.56)	*p* = 0.082
**Differences**	*p* < 0.001	*p* = 0.234	*p* = 0.062	*p* = 0.935	*p* = 0.935	

*Quantitative variables expressed as mean (SD) and range (minimum - maximum)*

† *Student’s t-test for two independent samples*

However, early-phase SUV ratio were significantly higher in pelvic lymph node metastases compared to prostate lesions (61.3 vs. 12.3; *p* < 0.001), with no difference found between the two groups in delayed-phase SUV ratio.

SUVmax was significantly higher on delayed-phase PET/CT compared to early-phase PET/CT in both prostate lesions (SUVmax delay 17.9 vs. SUVmax early 12.75; *p* = 0.001) and pelvic lymph nodes (SUVmax delay 32.9 vs. SUVmax early 26.35; *p* < 0.001).

Likewise, significantly higher SUV ratio was observed in the delayed phase compared to the early phase in prostate lesions (SUV ratio delay 21.9 vs. SUV ratio early 12.3, *p* < 0.001), with no statistically significant differences found in pelvic lymph nodes.

The reference standard was assessed in 52 patients (histopathology = 1; MRI = 1; CT = 14; bone scan = 7; PSMA-PET = 18; PSA after treatment = 32; follow-up PSA = 7), classifying the initial suspicious lesion as malignant in 75% of patients (*n* = 39) and as benign in 25% (*n* = 13).

For malignant lesions, higher delayed-phase SUVmax, delayed-phase SUV ratio, ΔSUVmax and IR were observed, without these differences being statistically significant (*p* > 0.05). The SUVmax and the different SUV ratios according to the lesion classified as benign and malignant are detailed in Table 3.

**Table 3:** Maximum SUV values (SUVmax) and SUV coefficients as a function of the lesion classified as benign and malignant according to the reference standard

	Benign lesions (n = 13)				Malignant lesions (n = 39)				Asymptotic sig. (bilateral)
	median	IQR	max	min	median	IQR	max	min	
SUVmax early	9,54	21,3	63,8	1,8	8,8	11,8	169	1,33	0,96
SUVmax delay	7,3	15,9	66,5	1,9	9,2	13,9	143,2	1,6	0,739
SUV ratio early	11,36	36,2	123,1	1,6	11,96	14,7	183	1,8	0,46
SUV ratio delay	11,1	32,6	121,9	2,9	17	23	179,4	1	0,337
△SUVmax	3	15,03	77,7	-7,7	3,7	3,7	29,1	-0,4	0,492
IR (%)	30,6	84,9	171	-331,9	31,83	31,83	912,3	-2,97	0,209
SUVratio early/delay	1,31	0,83	2,71	-0,33	1,3	1,3	3,8	0,97	0,369

*IQR = Interquartile range*

Additional delayed pelvic PET/CT imaging as part of the PSMA-PET procedure resulted in a change in molecular TNM staging in 22 out of 136 patients (16.2%). The highest percentage change was obtained in the miN classification (14/22 patients, 63.6%), followed by the miT classification (7/22; 31.8%) and lastly miM (1/22 patients; 4.5%).

**Fig. 1:**
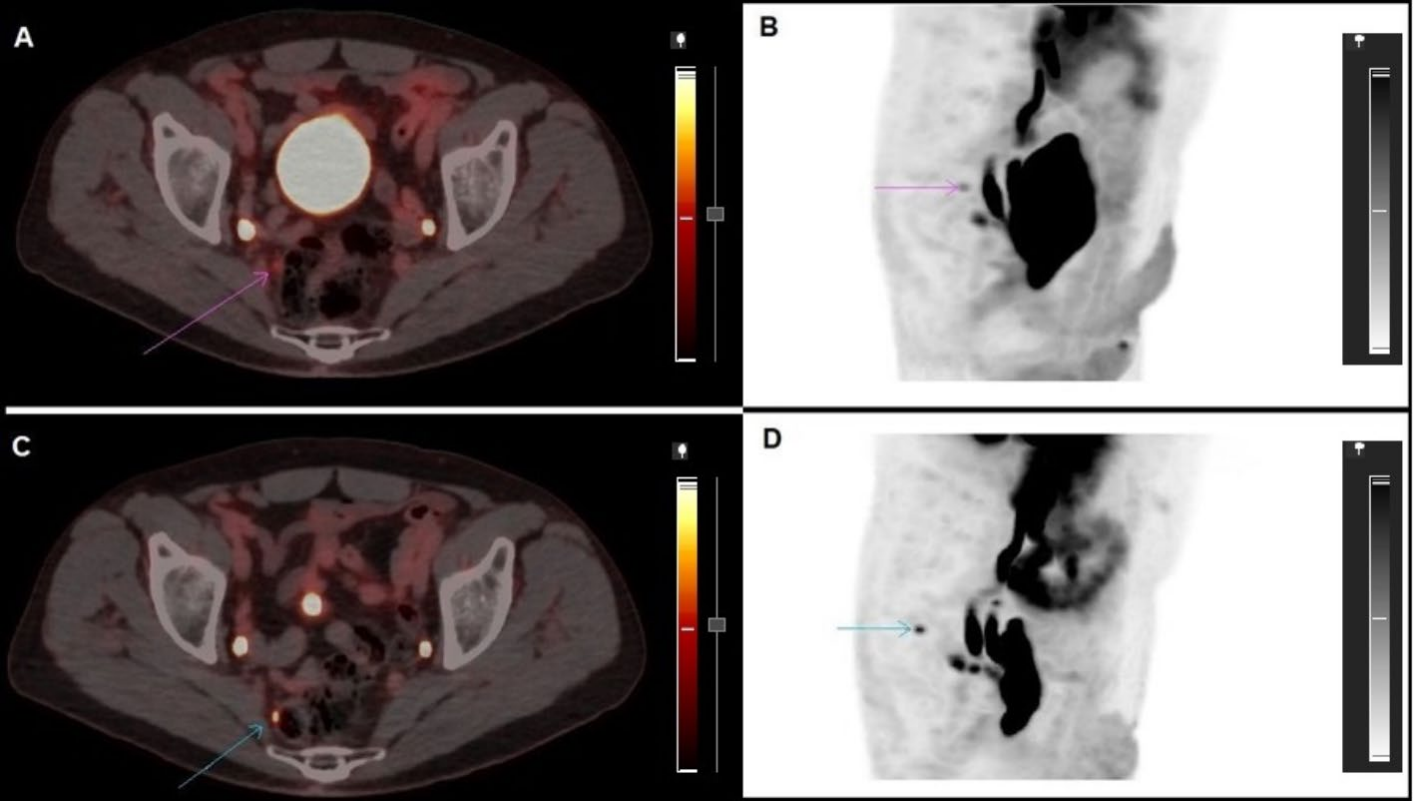
**A** and **B**) Axial views and MIP of the early phase PET-PSMA showing a small right pararectal lymph node with mild PSMA expression of uncertain etiology. **C** and **D**) Second-stage axial and MIP images showing a moderate increase in PSMA expression in this lymph node and suggesting malignancy. In this patient, the PSA level decreased after RT administration in the referred lymphatic regions

As for the patients in whom miN was modified (14/22), in most cases (8/14), the late miN was re-staged from miN0 to miN1: in 6/8 due to increased uptake in doubtful nodes, as can be seen in Fig. 1, and in 2/8 due to an increase in the number of pelvic lymph nodes. In 3/14 patients new lymph nodes appeared in the delayed image that changed the miN1 to N2. In 3/14 patients the degree of uptake of the lymph nodes visualised in the early image decreased, which meant a change from miN1 to miN0.

In those patients in whom the miT was modified (7/22 patients), in 5/7 the local recurrence was appropriately categorised due to delayed imaging from miT0 to miTr. In most cases (4/5) a doubtful deposit was evident on early imaging, but could be confirmed by the second stage scan, while in the other case the focus was new onset. In 2/7 patients the suspicious focus in the prostate on early imaging disappeared on delayed imaging, downstaging from miTr to T0.

**Fig. 2:**
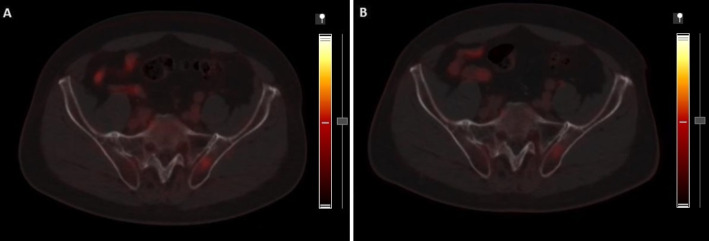
**(A)** Axial section of PET-PSMA in early phase showing a focal increase in PSMA expression in the left ilium bone (SUVmax 3.7), with no underlying morphological lesion and of doubtful pathological value. **(B)** Same axial section in second-stage showing decreased PSMA expression (SUVmax 2.4), therefore indicating a nonspecific finding

The only patient in whom the delayed image led to a change in the staging of bone involvement, the degree of uptake of a pelvic lesion decreased, as can be seen in Fig. 2, and was then downgraded from miM1b to miM0, bearing in mind, moreover, that there were no other hyperintense bone lesions in the rest of the skeleton.

Finally, we found that patients in whom delayed pelvic imaging resulted in a change in molecular TNM classification were significantly older (70.32 ± 7.3 vs. 65.7 ± 6.32 years; *p* = 0.006) and had a higher PSA level (1.14 ± 1.2 ng/ml vs. 0.69 ± 0.75 ng/ml; *p* = 0.024) than those in whom delayed imaging did not provide additional information (Table 4).

**Table 4:** Differences in baseline patient characteristics according to whether delayed pelvic PET/CT imaging had diagnostic benefit in terms of change in miTNM staging (no change/change in miTNM classification)

	No change (*n* = 113)	Yes change (*n* = 22)	*P*-value
Age (years)*	65.7 (6.32) (49–78)	70.32 (7.3) (56–84)	0.006 †
PSA (ng/ml)**	0.69 (0.75) (0.01–3.7)	1.14 (1.2) (0.25–4.29)	0.024 ¥
**Gleason (%**,** n)**			0.816 **‡**
**4**	0% (0)	4.5% (1)	
**5**	1.8% (2)	0% (0)	
**6**	23% (26)	18.2% (4)	
**7**	48.7% (55)	50% (11)	
**8**	17.7% (20)	18.2% (4)	
**9**	8% (9)	9.1% (2)	
**10**	0.9% (1)	0% (0)	

*Quantitative variables expressed by mean (SD) and range (minimum - maximum). ** Quantitative variables expressed as median (IQR) and range (minimum - maximum)

*†Student’s t-test for two independent samples*

*¥ U-Mann Whitney test*

*‡ Chi-square test for linear trend*

## Discussion

In this study we retrospectively analysed 136 patients who underwent dual-phase PSMA-PET with [^18^F]DCFPYL. Most of the patients (91.2%) were referred to our unit for biochemical recurrence with PSA value between 0.2 and 2 ng/ml (approved indication). Therefore, in these patients, the tumour burden to be expected is low, with a small number of lesions, which may also be small in size and/or with a low degree of uptake of PSMA, which could generate diagnostic doubts. For this reason, dual imaging may help to clarify these doubtful lesions. This is due to the continued accumulation of PSMA in prostate cancer lesions, leading to increased detectability of small lesions ((Schmuck et al. 2017a, b). 

In our group of patients there was a high percentage of negative studies (46.3%). In the remaining 73 patients 141 lesions were found, with a change in the miTNM after delayed imaging in 22 patients, i.e. 16.2% of the total.

In 7 of the 22 patients in whom the miT was changed, in one case a lesion was detected on delayed imaging that was not visible on early imaging. This patient showed a decrease in PSA following radiotherapy after the PET study. In two other cases, the early imaging findings were defined as non-malignant lesions due to the decreased SUVmax on delayed imaging. In one of these cases, a subsequent PSMA-PET was negative, confirming the non-malignant nature of the finding. Finally, in the remaining four cases, doubtful images were evident on early imaging due to low uptake, which were considered malignant lesions due to the increased SUVmax. Of these 4 patients, 3 patients received RT and there was a decrease in PSA in 2 of them and in one patient who did not receive treatment, PSA was increased in subsequent controls.

Regarding miN, there were 14 patients in whom delayed imaging had an impact on patient classification. Three patients showed a lymph node with low PSMA uptake on early imaging and decreased SUVmax on delayed imaging and were therefore classified as non-malignant. Subsequent follow-up showed a decrease in PSA without any treatment. However, in 6 other cases, the increase in SUVmax between early and delayed imaging allowed the detected lymph nodes to be classified as malignant. After treatment, a decrease in PSA was observed in these patients. In addition, five other patients had hyperenhancing lymph nodes detected on delayed imaging that had not been identified on early imaging, with three patients going from miN0 to miN1 and two patients from miN1 to miN2.

Finally, in miM, in one patient with a doubtful image due to its low degree of uptake, SUVmax decreased in the delayed imaging, and was therefore considered non-malignant.

As in other studies, in our sample of patients we also found more lesions in the delayed imaging than in the early imaging, being mainly in the pelvic lymphatic involvement where the delayed imaging contributed most, corresponding to 63.6% of the changes found ((Alberts et al. 2021; Afshar-Oromieh et al. 2017).

In 16.2% of patients, there was a change in the miTNM between early imaging and delayed imaging. This value is above other authors, such as Wondergem et al. ((Wondergem et al. 2017), who found a 9.2% change in TNM. However, it is much lower than those found in other studies such as that of ((Librizzi et al. 2023), who found a change in TNM of 66.7%, although the greatest number of changes also occurred in lymphatic involvement (55.6% vs. 63.6%).

The value of delayed imaging has been extensively studied in PET studies of oncological patients, fundamentally in FDG studies, demonstrating an increase in sensitivity and specificity due to the fact that the progressive increase in uptake by malignant lesions and the decrease in radiotracer in the rest of the tissues leads to an increase in the lesion/background ratio. The results of these studies support the inclusion of delayed imaging, as it not only detects lesions not evident in the baseline image, but also allows the dynamics of uptake by the lesion detected in the initial image to be assessed. Several authors have analysed the value of delayed imaging in patients with biochemical recurrence of prostate cancer using different radiotracers ([^68^Ga]PSMA, [^18^F]PSMA, [^18^F]DCFPYL). International guidelines recommend acquisition of an image around 60 min post-injection, but delayed imaging is under discussion, as well as the best time for acquisition (90 min p.i., 120 min p.i. and even 180 min p.i.). Schmuck et al.^17^ found that only 3.4% of lesions suggestive of disease recurrence were detected at 3 h post-injection.

In our study, a baseline image was systematically performed at 60 min and a delayed imaging at 120 min post-injection. Given that, in most cases, the second-stage imaging clarified the etiology of a doubtful uptake deposit pre-existing in the early imaging, and the increased radiation dose involved in delayed imaging CT and equipment workloads, delayed imaging should probably be reserved for selected cases. In our study, the patients who benefited from delayed imaging were those who were older and had a higher PSA level, without finding a statistical relationship with the Gleason score.

Thus, further prospective studies should be carried out with a larger number of patients to confirm these results and, if confirmed, reserve dual imaging for this group of patients, which would avoid unnecessarily prolonged stays in PET units for a large number of patients and would lighten the workload of the teams.

Our study includes a number of limitations that need to be mentioned. Firstly, it is a single-centre, retrospective study. Secondly, a reference standard was only available in 38.2% of the patients, and we were unable to use the same gold standard in all cases, which could significantly affect the reproducibility of the results. In addition, most of the suspicious lesions did not have histopathological confirmation, although clinical-radiological follow-up is a well-established reference standard. Thirdly, most of the patients analysed were patients with biochemical recurrence, so the role of this dual imaging in the initial staging of patients diagnosed with prostate cancer cannot be assessed.

## Conclusions

In conclusion, pelvic delayed imaging in patients with biochemical recurrence of prostate cancer undergoing PET/CT with [^18^F]DCFPYL shows a non-negligible influence on patient staging, modifying the miTNM classification in 16.2% of cases, with pelvic lymphatic involvement benefiting the most from the dual study.

Older patients with higher PSA are the group of patients in whom late acquisition provides the most additional information.

## Electronic supplementary material

Below is the link to the electronic supplementary material.

Supplementary Material 1

Supplementary Material 2

## Declarations

### Guarantor

The scientific guarantor of this publication is Rosa María Álvarez Pérez.

### Statistics and biometry

Miriam Guerra Gómez has significant statistical expertise.

### Study subjects or cohorts overlap

No subject subjects or cohort overlap reported.
