# Supplementary material for: Impact of delayed pelvic imaging on the staging of biochemical recurrence in prostate cancer patients using [18F]DCFPYL PET/CT: a retrospective evaluation
Source: EJNMMI Rep. 2025 Feb 1;9(1):5. doi: 10.1186/s41824-025-00238-8 (PMC11785851; doi:10.1186/s41824-025-00238-8)
Supplement: Supplementary file 1 [file 41824_2025_238_MOESM1_ESM.odt]

IMPACT OF DELAYED PELVIC IMAGING ON THE STAGING OF BIOCHEMICAL RECURRENCE IN PROSTATE CANCER PATIENTS USING [18F]DCFPYL PET/CT: A RETROSPECTIVE EVALUATION

Authors: Rodríguez-Pajuelo, Ana 1*; Guerra-Gómez, Miriam 1*; Cuenca-Cuenca, Juan Ignacio1 ; Freire-Macías, José María1 ; Jiménez-Hoyuela García, José Manuel1, Álvarez-Pérez, Rosa María1.

* Ana Rodríguez Pajuelo and Miriam Guerra Gómez are considered the first authors of this manuscript.

Affiliation:

1 Nuclear Medicine Department. University Hospital Virgen del Rocío, Seville.

*Corresponding author:

- Ana Rodríguez Pajuelo.

E-mail: [anarodriguezp1511@gmail.com](mailto:anarodriguezp1511@gmail.com)

ORCID: 0009-0002-1219-1847

Ph: +34 660375281
